# Structure and Metabolic Characteristics of Intestinal Microbiota in Tibetan and Han Populations of Qinghai-Tibet Plateau and Associated Influencing Factors

**DOI:** 10.3390/microorganisms11112655

**Published:** 2023-10-28

**Authors:** Jin Lv, Ping Qi, Xiangdong Yan, Liuhui Bai, Lei Zhang

**Affiliations:** 1 The First Clinical Medical College, Lanzhou University, Lanzhou 730000, China; lvj21@lzu.edu.cn (J.L.); qip21@lzu.edu.cn (P.Q.); yanxd21@lzu.edu.cn (X.Y.); bailh21@lzu.edu.cn (L.B.); 2 Department of General Surgery, The First Hospital of Lanzhou University, Lanzhou 730000, China; 3 Key Laboratory of Biotherapy and Regenerative Medicine of Gansu Province, The First Hospital of Lanzhou University, Lanzhou 730000, China

**Keywords:** Qinghai-Tibet Plateau, intestinal microorganisms, Tibetans, Han nationality, metabolomics

## Abstract

Residents of the Qinghai-Tibet Plateau might experience shifts in their gut microbiota composition as a result of the plateau environment. For example, high altitudes can increase the abundance of obligate anaerobic bacteria, decrease the number of aerobic bacteria and facultative anaerobic bacteria, increase probiotics, and decrease pathogenic bacteria. This study aimed to determine the structure and metabolic differences in intestinal microbial communities among the Tibetan and Han populations on the Qinghai-Xizang Plateau and shed light on the factors that influence the abundance of the microbial communities in the gut. The structural characteristics of intestinal microorganisms were detected from blood and fecal samples using 16S rRNA sequencing. Metabolic characteristics were detected using gas chromatography–time-of-flight mass spectrometry (GC–TOFMS). The influencing factors were analyzed using Spearman’s correlation analysis. Bacteroides and Bifidobacterium were dominant in the intestinal tract of the Han population, while Bacteroides and Prevotella were dominant in that of the Tibetan population, with marked differences in Pseudomonas, Prevotella, and other genera. Ferulic acid and 4-methylcatechol were the main differential metabolites between the Tibetan and Han ethnic groups. This may be the reason for the different adaptability of Tibetan and Han nationalities to the plateau. Alanine aminotransferase and uric acid also have a high correlation with different bacteria and metabolites, which may play a role. These results reveal notable disparities in the compositions and metabolic characteristics of gut microbial communities in the Tibetan and Han people residing on the Qinghai-Tibet Plateau and may provide insights regarding the mechanism of plateau adaptability.

## 1. Introduction

The Qinghai-Tibet Plateau is a unique ecosystem characterized by extreme cold, low oxygen, and high ultraviolet indices, contributing to comprehensive and profound effects on the human body [1]. The plateau environment can cause headaches, chest congestion, shortness of breath, nausea and vomiting, and increased blood pressure, in addition to diseases of the heart, brain, lungs, and other systems. Most people are affected by the cold, gastrointestinal discomfort caused by diarrhea, and infections that may harm human health [2,3]. Recently, the effect of the plateau environment on the human intestine has been a focus of high-altitude medicine research. Human body stimulation under conditions of hypoxia, extreme cold, and other factors can cause intestinal mucosal ischemia and damage to the intestinal barrier, which leads to imbalanced intestinal microbiota. In addition, living at a high altitude for extended periods can induce adaptive alterations in the gut microbiota composition [4]. The human intestinal tract harbors an extensive community of countless microorganisms, often referred to as the “invisible organs,” which play vital roles and closely interact with our physiology. The structural and operational integrity of these microorganisms is crucial for forming an intestinal barrier that aids nutrient digestion and absorption and ensures a balance in immune and metabolic functions [5,6].

The Qinghai-Tibet Plateau in China is inhabited by numerous diverse ethnic communities, of which, the predominant populations are the Tibetan and Han. The Han population live in a high-altitude environment; they have experienced a series of chronic pathological changes, and the functions of multiple organs have gradually decreased in relation to hypoxia [2]. Headaches, insomnia, loss of appetite, and other uncomfortable symptoms frequently occur in this population, in addition to chest tightness, shortness of breath, and fatigue that appear in serious cases [3]. In contrast, Tibetans residing on the plateau exhibit exceptional adaptability to their high-altitude surroundings; their lungs grow faster during adolescence and become larger in size and volume in adulthood. They also have an increased forced vital capacity and forced expiratory volume in 1 s, which increases the surface area of the lungs to enable gaseous exchange [7]. In addition, blood flow and the capillary density of their muscle tissue are higher, which increases blood perfusion and oxygen delivery [8,9]. EPAS1 and other genes work in conjunction to adjust oxygen supply and hemoglobin regulation, which contribute to the Tibetan people’s ability to thrive in the challenging plateau conditions [10,11].

Extensive evidence has revealed that the intestinal microbial communities of human ethnicities differ [12]. To this regard, the richness and diversity of intestinal microorganisms is greater in the Tibetans than in Han individuals. For example, the gut microbiota of Tibetans tends to be abundant in *Blautia*, *Butyricimonas*, *Clostridium*, and *Desulfovibrio* [13]. However, at four different altitude gradients, the gut microbiota of Han individuals contains higher levels of Bacteroides, Faecalibacterium, and Megamonas compared with that of Tibetan individuals [14].

Numerous studies have conducted in-depth research on the human intestinal microbiota at high altitude; however, they are often limited to a single microbial group. In recent years, with the development of metabolomic technology, we can elucidate the influence of the plateau environment on the metabolic function of human intestinal microbiota. By comprehensively analyzing the composition and metabolites of intestinal microbiota, we can fully understand the potential impact of the plateau environment on human health.

We discuss changes in intestinal microbial composition when the same population group is exposed to different conditions over time and provide a theoretical basis for subsequent research and development of intestinal microbial-mediated prevention and treatment of altitude sickness.

## 2. Materials and Methods

### 2.1. Study Cohort

The Qinghai-Tibet Plateau is situated in the northwestern part of China at an average elevation of >3000 m. Tibetan (*n* = 45) and Han Chinese (*n* = 41) participants were randomly selected from the Qinghai-Tibet Plateau based on the inclusion criteria: they had not experienced (1) severe digestive system diseases in the past, (2) recent digestive system discomfort or disease, or (3) diseases or infections in the past period of time. In addition, they could (4) not have recently used antibiotics, immunosuppressive agents, or intestinal microecologics. Furthermore, (5) females in the study could not be pregnant or lactating and could not be undergoing menstruation. The following exclusion criteria were set: (1) those clinically diagnosed with major intestinal diseases and chronic inflammation; (2) those taking aspirin, insulin, metformin, statins, or metoprolol; (3) those who had used antibiotics or probiotics in the past eight weeks; (4) and those who refused to sign the informed consent form.

Through rank sum test analysis, there is no statistical difference in baseline data such as age, gender, and Body mass index(BMI) between Tibetan and Han people (Table 1). This research was approved by the Ethics Committee at the First Hospital of Lanzhou University (approval number: LDYYLL2019-36), and all participants provided informed consent before participating in the study.

### 2.2. Sample Collection, Processing, and 16S rRNA Analysis

Blood and fecal samples were collected from each study participant: 200 mg of feces was collected in a clean container during normal defecation, and 8 mL of blood was drawn. There were no replicates. Immediately after collection, 2 mL of blood was separated using centrifugation to obtain the serum, and the remaining 6 mL was used for biochemical marker testing. The serum and fresh fecal samples were immediately placed in the refrigerator and shipped to the laboratory for processing, where 200 mg of feces and 1 mL of serum were placed into two separate sterile 2 mL centrifuge tubes. 

### 2.3. DNA Extraction

Samples were frozen immediately after collection and kept at −80 °C until extraction. The total genomic DNA was isolated utilizing the FastDNA^®^ SPIN Kit for soil (MP Biomedicals, Santa Ana, CA, USA), according to the manufacturer’s instructions. Agarose gel electrophoresis was conducted to check the genomic DNA integrity. The Nanodrop 2000(10x Genomics, Madison, NY, USA) and Qubit3.0 spectrophotometer(Thermo Fisher Scientific, Carlsbad, CA, USA) were employed to quantify DNA concentration and purity.

### 2.4. Sequencing

The V3–V4 hypervariable regions of the 16S rRNA gene and spike-ins were amplified with the following primers: 341F (5′-CCTACGGGNGGCWGCAG-3′) and 805R (5′-GACTACHVGGGTATCTAATCC-3′). The amplicons were then sequenced using an Illumina NovaSeq 6000 sequencer(Illumina, San Diego, C, USA). After agarose gel electrophoresis, a mixture was formed by combining three parallel amplification products sourced from the same specimen. An Agencourt am pure XP kit (Beckman Coulter, Brea, CA, USA) was used to purify the samples. Using a primer containing an index sequence, a unique tag sequence was introduced at the library’s end using high-fidelity PCR so that a plurality of samples could be mixed during sequencing on a downstream computer. Sample differentiation using different tag sequences was then achieved in bioinformatic processing. A raw library was obtained after repeated testing and purification. The concentration of the sample library was determined, and it already possessed specific index tags. The sample library was appropriately diluted according to the above quantitative results, and the library was labeled with the corresponding index tag. These libraries were then accurately quantified by Qubit (Thermo Scientific, Waltham, MA, USA) and mixed in a molar ratio of 1:1. An Agilent 2100 Bioanalyzer(Agilent Technologies, Santa Clara, CA, USA) was used to examine the insert size of the sequencing library within the pooled library. Non-specific amplification was confirmed between 120 and 200 bp, along with the concentration of the sequencing library. The library was sequenced using the two-terminal sequencing strategy of the NovaSeq 6000 platform, SP-Xp (PE250).

### 2.5. Illumina Read Data Processing and Analysis

The raw read sequences were processed in QIIME2 [15]. The adaptor and primer sequences were trimmed using the cutadapt plugin. The DADA2 plugin was used for quality control and to identify amplicon sequence variants (ASVs) [16]. The “filterAndTrim” method was utilized with the parameter “maxEE = 2” to eliminate expected errors in paired-end reads. The “learnErrors” function was applied to perform quality correction. Following quality control, an ASV table was generated. Finally, to enhance data purity, chimeric sequences were removed.

Taxonomic assignments of ASV representative sequences were performed with a confidence threshold of 0.8 by a pre-trained Naive Bayes classifier that was trained on the Greengenes (version 13.8). The spike-in sequences were then identified and the reads counted. A standard curve for each sample was generated based on the read counts versus spike-in copy number, and the absolute copy number of each ASV in each sample was calculated by using the read counts of the corresponding ASV. Since the spike-in sequence was not a component of the sample microbiota, the spike-in sequence needed to be removed in the subsequent analysis [17].

### 2.6. Non-Targeted Fecal Metabolomics

A 50 ± 1 mg fecal sample was placed in 2 mL tube, and 1000 μL of a pre-cooled extract (3:1 *v*/*v* methanol:chloroform volume ratio = 3:1) was added. Next, 10 μL ribitol was added, and the blend was vortexed for 30 s. Steel balls were also added to aid homogenization. A 45 Hz grinder was used for 4 min, and the sample was subjected to ultrasonic treatment for 5 min (in an ice-water bath), which was repeated thrice. The specimen was then centrifuged at 13,400 relative centrifugal force (RCF) at 4 °C for 15 min. The solution was pipetted (150 µL) from the solution into 1.5 mL tubes. Samples were then collected (100 µL) and blended to generate quality control (QC) samples. Next, the sample underwent drying in a vacuum concentrator, after which, 30 μL methoxyamine salt reagent (methoxyamine hydrochloride dissolved in pyridine at 20 mg/mL) was added, mixed gently, and placed in an oven at 80 °C for 30 min. Forty microliters of N,O-bis(trimethylsilyl)trifluoroacetamide supplemented with 1% trimethylchlorosilane (*v*/*v*) was added, and the mixture was incubated at 70 °C for 90 min. 

The sample was cooled down to 25 °C, and 5 μL of fatty acid methyl esters (dissolved in chloroform) was incorporated into the mixed samples. Random sequence testing was conducted on a computer.

An ADB-5MS capillary column was employed in the mass spectrometry system. The following parameters were employed: 1 μL sample volume; splitless front inlet mode; 3 mL min^−1^ Front Inlet Septum Purge Flow 3 mL min^−1^, helium Carrier Gas Helium, DB-5 MS (30 m × 250 μm × 0.25 μm) Column DB-5 MS (30 m × 250 μm × 0.25 μm), 1 mL min^−1^. Column flow rate: 1 mL min^−1^, and oven temperature ramp: held at 50 °C for 1 min, raised to 310 °C at a rate of 10 °C min^−1^, and held at 310 °C for 8 min. Front injection temperature: 280 °C, Transfer Line Temperature: 280 °C, Ion Source Temperature: 250 °C, ionization energy: −70 eV, Mass range *m*/*z*: 50–500 *m*/*z*, acquisition rate: 12.5 spectra per second, and solvent delay: 6.27 min. 

MS-DIAL software (http://prime.psc.riken.jp/compms/msdial/main.html, 1 July 2023) was used for peak extraction, baseline correction, deconvolution, peak integration, and peak alignment analysis of the mass spectral data. The FiehnBinbase database (including mass spectral matching and retention time index matching) was used for substance qualification. Finally, peaks displaying a detection rate of <50% or an RSD of >30% in the QC samples were excluded.

### 2.7. Untargeted Serum Metabolomics

Samples (100 µL) were placed into 1.5 mL tubes and a pre-chilled extract (methanol with ribitol, 410 µL) was added to the samples. Samples were vortexed for 30 s followed by ultrasonication for 10 min (in an ice-water bath). The remaining conditions were the same as those described above.

### 2.8. Statistical Analyses

Statistical evaluations for group comparisons were executed using SPSS 22.0 for Windows (IBM, Chicago, IL, USA), and an independent Student’s *t*-test and the Mann–Whitney test were used. Correlations were determined through Spearman’s correlation. The resulting *p*-values were adjusted using the Benjamini–Hochberg false discovery rate (FDR) correction. Only FDR-corrected *p*-values below 0.05 were considered significant.

Microbiota analysis: 16S data were analyzed using Studio 4.2.3 [18]. The diversity index is obtained by the alpha diversity analysis method. A partial least squares-discriminant analysis (PLS-DA) was used, and R-package mixOmics was used to investigate differences between the community compositions of samples.

Metabolomics analysis: A multivariate statistical technique was applied for data analysis. The card value standards were a *p*-value < 0.05 (Student’s *t*-test) and the variable projection importance (VIP) of the first principal component of the OPLS-DA model with a *p*-value above 1.

Correlation analysis: Utilizing the pandas package 1.2.4,the matplotlib package 3.5.1 and networkx package 3.1 in Python 3.9.13, an investigation was conducted into the relationship among microbiota, metabolomics, and hematological parameters. Spearman’s correlations between the microbiota and variables in the clinical data were co-calculated. To show the relationships between the variables, an interaction network diagram was generated using the Plotly package.

## 3. Results

### 3.1. Alpha Diversity Analysis

The alpha diversity in the Tibetan and Han populations differed significantly, as indicated by multiple indices (Table 2). The species richness of Tibetan intestinal microbiota was higher, and there were more bacterial species. The observed index directly reflects the observed species abundance.

The overall uniformity of Tibetan intestinal microbiota was better than that of the Han individuals. Chao1 and the ACE index reflected species richness.

**Figure 1:**
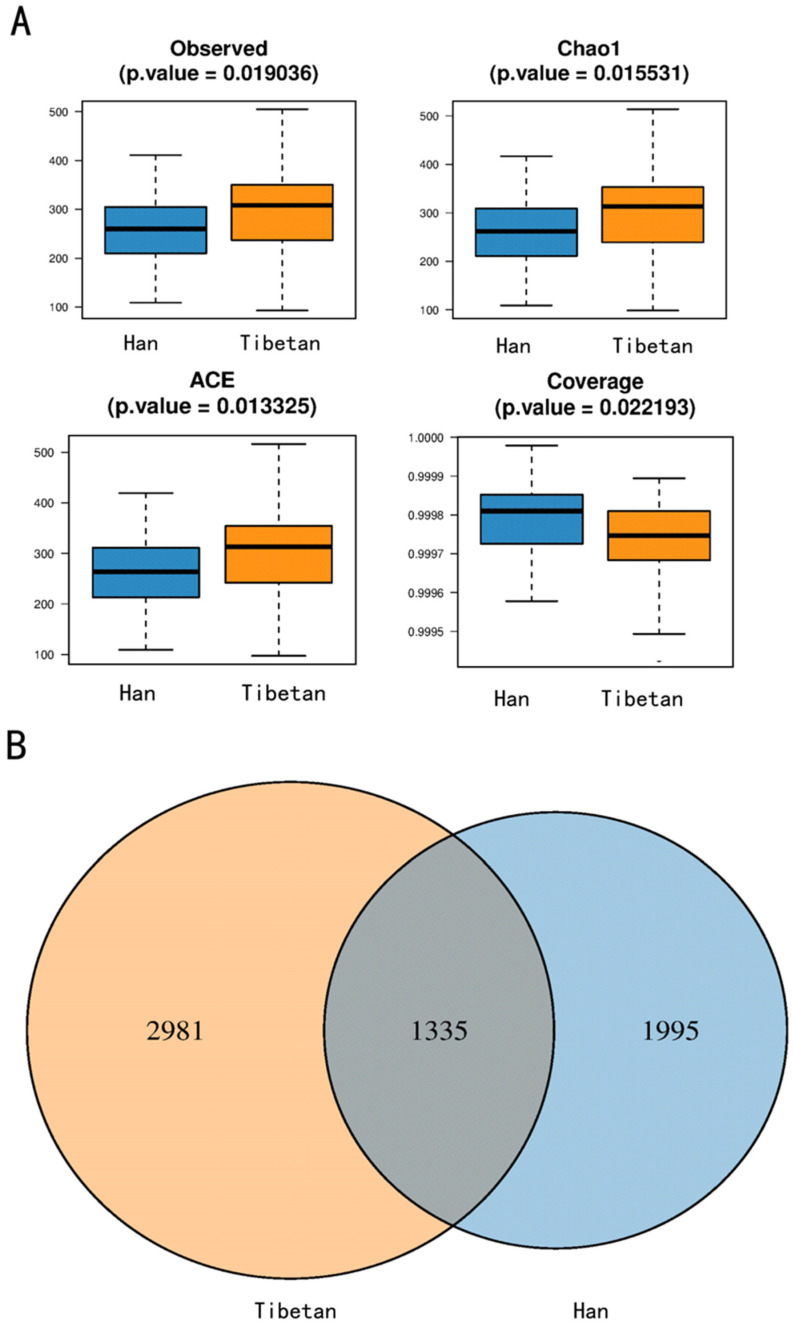
α diversity analysis. (**A**) Differential analysis of inter-group diversity indices based on Kruskal–Wallis rank sum test with p < 0.05 defined as the significant difference screening threshold and the multiple hypothesis testing correction for the p-value using Bonferroni’s method. Horizontal coordinate indicates different groups, while vertical coordinate indicates the diversity index values of sample communities in this group. Different groups are distinguished using different colors. (**B**) Screening for operational taxonomic units (OTUs) specific to each group of samples and for common OTUs between groups that were grouped into units. Different sample groups are represented by different colors, and the areas where the circles of different colors overlap are marked with numbers to indicate the number of common species.

This implied that the intestinal microbial community in the Tibetan population was comparatively more stable, and its function was more abundant than in the Han population. The coverage index of >0.9 indicated that the microbial community of samples was relatively complete and that it could be considered highly representative of the community structure and function (Figure 1A).

A total of 1335 identical operational taxonomic units (OTUs) were identified in the Tibetan and Han individuals. In contrast, 2981 and 1995 unique OTUs were found in the Tibetan and Han populations, respectively (Figure 1B).

### 3.2. Beta Diversity Analysis

**Figure 2:**
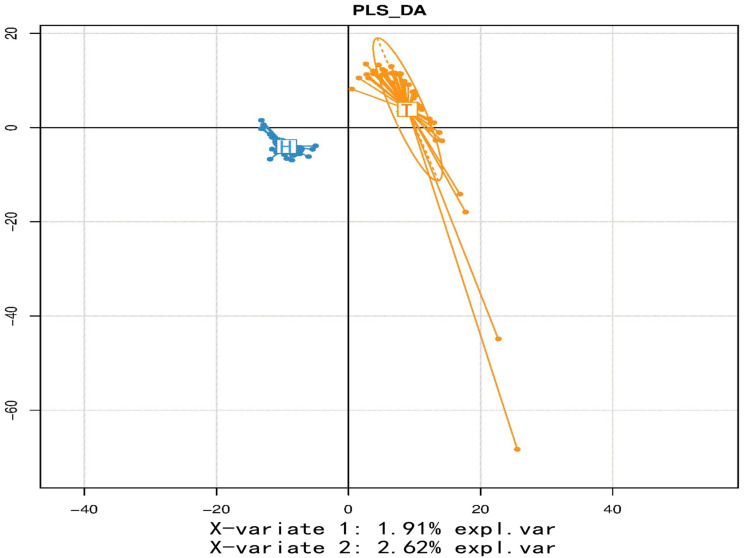
Inter−group differences in β diversity. Partial least squares-discriminant analysis (PLS−DA) possesses the capability of efficiently differentiating the measured data points across groups by appropriate rotation of the principal components and is used to identify influencing variables that can lead to the differences between groups. Different groups of samples are represented by different colors.

Beta diversity showed the variation in species diversity across distinct samples, and the PLS-DA showed notable distinctions in the structure of intestinal microbial communities of the two populations (Figure 2).

### 3.3. Microbial Community Analysis

**Figure 3:**
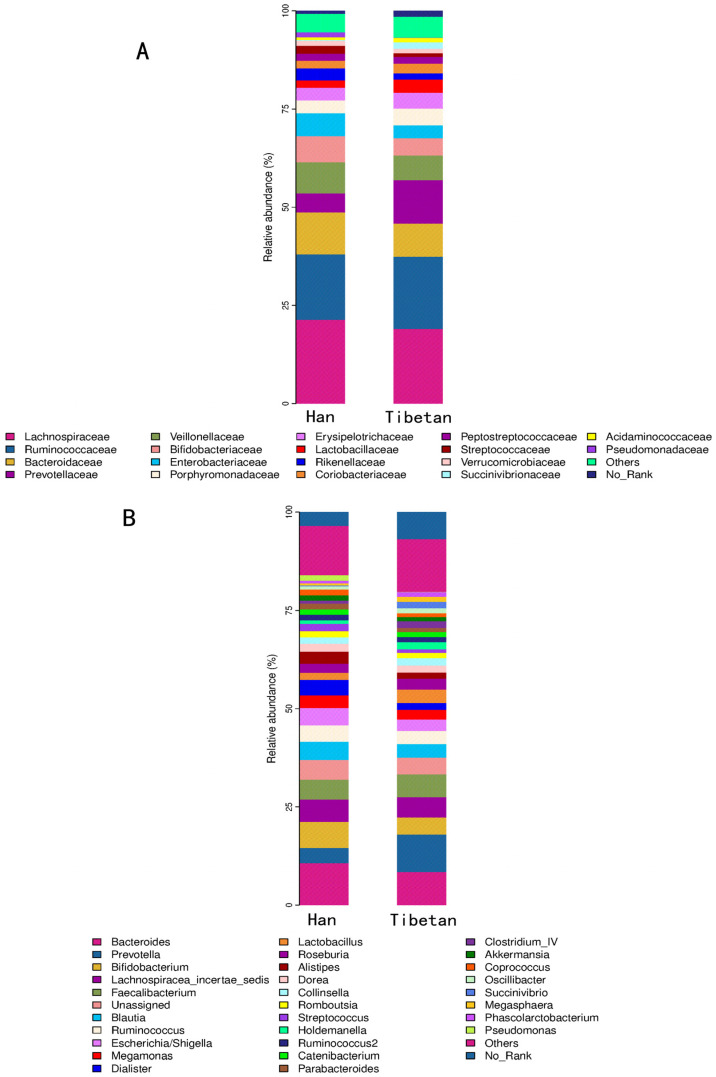
Inter-group differences in genus and family levels. Histograms of the species composition of the Tibetan and Han individuals at different classification levels are plotted. Species with a relative abundance of >1% per sample are plotted. (**A**) Differences in family level; (**B**) Differences in genus level.

The microbial communities were analyzed to understand the specific effects that living on the Qinghai-Tibet Plateau had on the intestinal microbial communities between the two populations. Microbial taxa occupying > 1% of the total abundance were selected from each sample to compile a histogram, and the structural compositions of the microbiotas of the two populations were determined (Figure 3). At the family level, the Han population harbored greater numbers of intestinal Pseudomonadaceae, Sutterellaceae, and Rikenellaceae, while the Tibetan population contained greater numbers of Prevotellaceae and Clostridiales_Incertae_Sedis_XIII.

With respect to the genus composition, the proportions of Pseudomonas, Parasutterella, Clostridium_XlVb, Alistipes, Anaerostipes, Butyricicoccus, Dialister, Coprococcus, and Clostridium_XVIII were greater in the Han population than in the Tibetan population. In contrast, Butyrivibrio, Clostridium_IV, Prevotella, and Christensenella were more prevalent in the Tibetan people than in the Han people.

**Figure 4:**
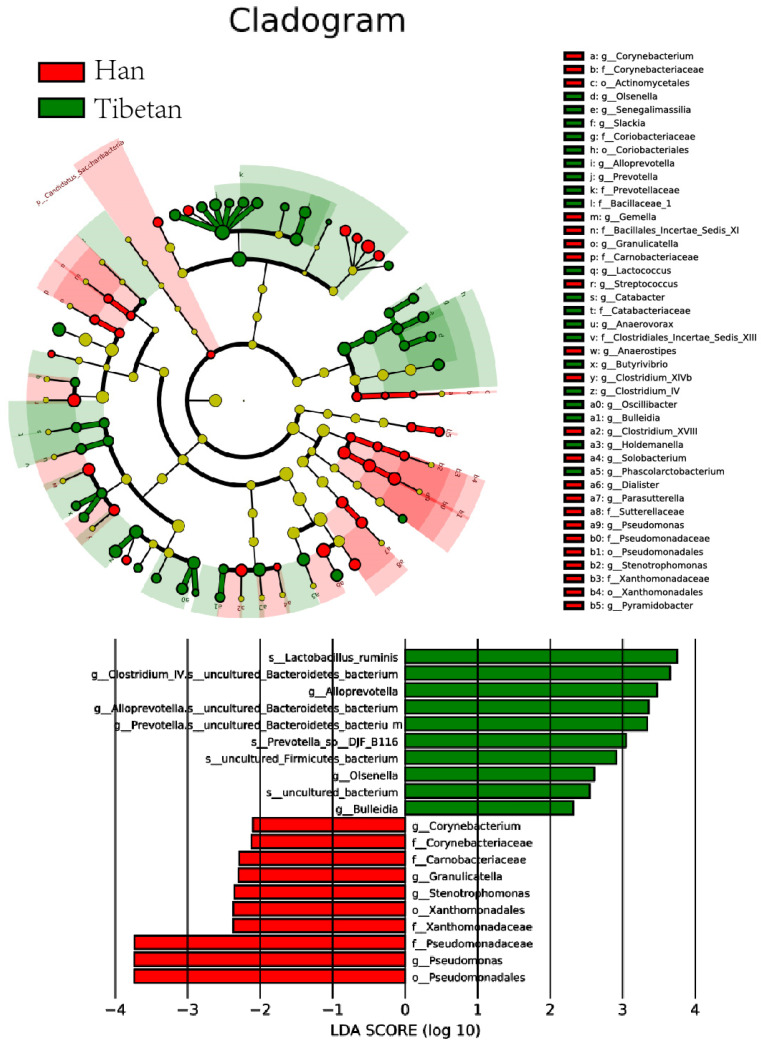
Potential biomarkers of inter-group differences. The first 50 species with the smallest p-values were selected to compile the evolution bifurcation diagram using |LDA| > 2 and the p-value to screen significant differences. First, 10 species with the smallest p-value in each group were selected to draw the bar graph shown here. Different species with an LDA score (log10) of >2 in different groups and significantly greater abundance in this group are represented by bars with different colors. Magnitude of the LDA score is denoted by the length of the corresponding bar chart. The length of the bar graph represents the size of the LDA score value. LDA, linear discriminant analysis.

The species considered most likely to explain the inter-group differences were selected using the linear discriminant analysis (LDA) effect magnitude method (LEfSe) as the difference screening threshold. These species were regarded as potential biomarkers for the two populations (Figure 4).

### 3.4. Metabolomics

Several metabolites were found to be present at relatively high levels in the intestinal tract of the Tibetan population, including ferulic acid, linoleic acid methyl ester, lyxose minor, galactose-6-phosphate 2, beta-hydroxymyristic acid, and pyrogallol. However, higher levels of methanol phosphate, ferulic acid, and linoleic acid methyl ester were found in the serum of the Tibetan.

**Figure 5:**
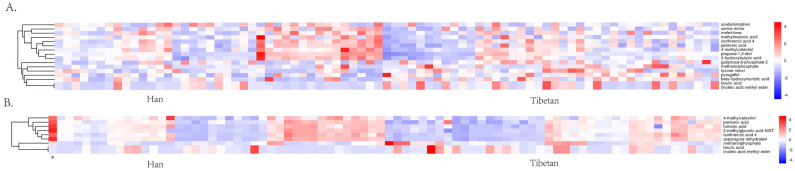
(**A**) Differential metabolites in feces. (**B**) Differential metabolites in serum. X−axis represents Tibetans and Han individuals, and Y-axis represents metabolites. Colored segments at various locations depict metabolite expression: red and blue indicate high and low substance content expression, respectively.

Numerous elevated metabolites were discovered in the gut of Han individuals dwelling at high elevations. These metabolites included serine minor, isothreonic acid 4, propane-1,3-diol, 4-methylcatechol, 3-hydroxybutyric acid, pentonic acid, and melezitose. Serum levels of isothreonic acid 4, 4-methylcatechol, asparagine dehydrated, fumaric acid, and 2-methylglyceric acid NIST were higher in Han individuals (Table 3). The discrete metabolites identified were then categorized via cluster analysis and visually depicted on a heat diagram (Figure 5).

### 3.5. Metabolic Pathways

**Figure 6:**
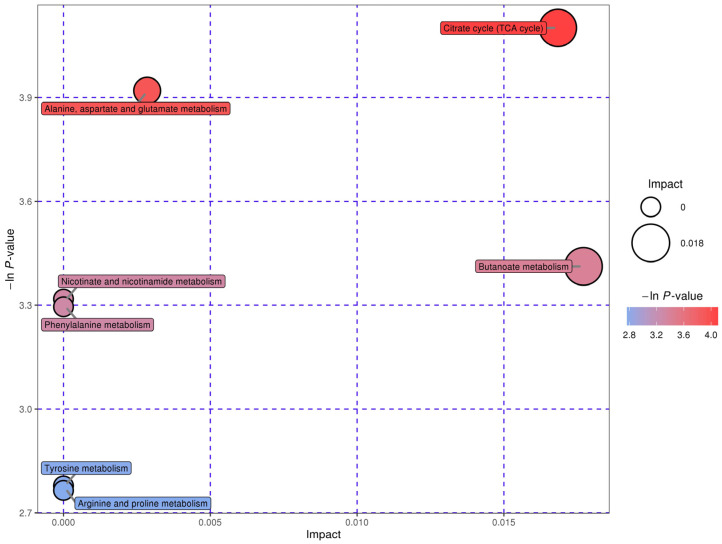
Inter-group differential metabolic pathways. Every bubble symbolizes a distinct pathway. X-axis of the bubble and the bubble’s scale reflect the importance of the pathway. A bigger bubble indicates a bigger influencing factor. The altitude where the bubble resides and its color communicated the *p*-value as revealed by enrichment analysis. The vertical coordinate where the bubble is located and the color of the bubble represent the *p*-value from the enrichment analysis (taking the negative natural log, i.e., the -ln *p*-value). The darker color is correlated with a smaller *p*-value and more significant enrichment. More profound coloring is associated with lesser *p*-values and enhanced enrichment significance.

Based on the screened metabolites, the Kyoto Encyclopedia of Genes and Genomes database was used to conduct a pathway enrichment analysis. The most superior level pathways were those of the tricarboxylic acid cycle, alanine, aspartate, and glutamate, butanoate, nicotinate and nicotinamide, phenylalanine, tyrosine, and arginine and proline metabolisms (Figure 6).

### 3.6. Hematology Parameter Analysis

**Figure 7:**
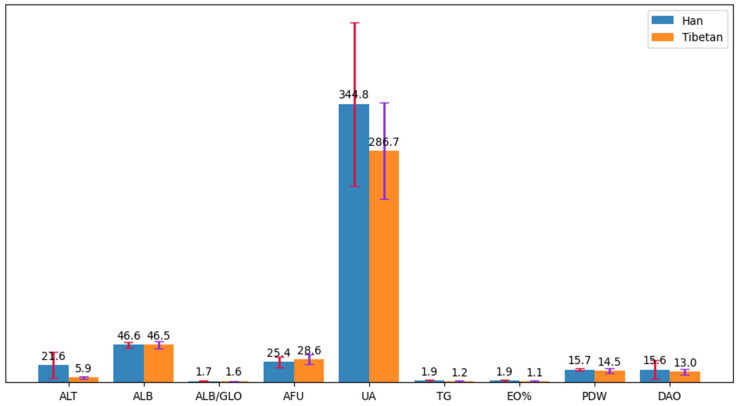
Hematology parameters of inter-group differences.

To assess alterations in the hematology parameters of the two population groups, the hematology parameters were first generated by blood biochemical assays and enzyme-linked immunosorbent assays. In the Han population, blood levels of alanine aminotransferase (ALT), Albumin(ALB), uric acid (UA), triglycerides (TG), and diamine oxidase (DAO) and the albumin/globulin Ratio (ALB/GLO), eosinophil percentage (EO%), and platelet distribution width were greater in the Han population than in the Tibetan cohort, while the blood level of Alpha-L-Fucosidase was statistically higher in the Tibetan cohort (*p* < 0.05) (Figure 7). 

### 3.7. Correlation Analysis

**Figure 8:**
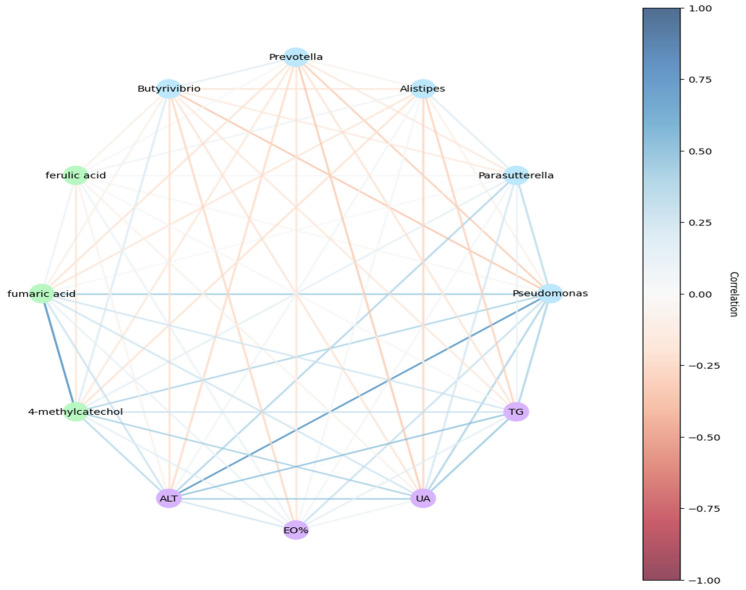
Relationship among key bacteria, characteristic metabolites, and hematology parameters. Blue node, representative microorganism; green node, differential metabolite; purple node, clinical index. Blue line between nodes indicates a positive correlation, while orange line indicates a negative correlation. Depth of the color indicates the correlation coefficient, while a darker line indicates a higher R value.

Representative microbial genera showing noteworthy differences were identified in the two populations, and metabolites at different concentrations and related clinical indicators were identified. Parasutterella and Pseudomonas were significantly increased in the Han population and showing a positive connection to serum ALT levels. Pseudomonas showed a positive connection to fumaric acid, 4-methylprednisolone, UA, and TG. In addition, elevated 4-methylprednisolone was positively correlated with ALT and UA in the Han population (Figure 8).

## 4. Discussion

We investigated and compared the intestinal microbial communities, hematological parameters, and metabolic characteristics of Tibetan and Han individuals in the Tibetan Plateau. The predominant intestinal microorganisms of the Han population were *Bacteroides*, while those of the Tibetan population were *Prevotella*. The gut microbiota composition varied between Tibetan and Han individuals; Tibetans exhibited greater richness and diversity indices in terms of their intestinal microorganisms than Han individuals, and these results align with the outcomes observed in prior research. Zhi long et al. also reported that the α and β diversity of intestinal microbiota in Tibetans is greater than that in the Han population [14]. 

The metabolic rate accelerates within a low-temperature plateau environment, and more energy is required to maintain body temperature and perform normal activities. The guts of the Tibetan population contained greater numbers of *Prevotella* and *Butyrivibrio*, which could have resulted in more short-chain fatty acids (SCFAs) [19,20] that provide large amounts of energy for the body. For example, butyric acid serves as the primary fuel for intestinal epithelial cells after oxidization in the mitochondria [21]. Ferulic acid, which is abundant in the intestines of Tibetan people, can regulate the differentiation and development of brown adipocytes by upregulating PGC-1α and PPARγ, which may have helped Tibetans to generate more heat on the cold Qinghai-Tibet Plateau [22,23].

Our study further reveals that the Han and Tibetan populations had high and low levels of blood DAO, respectively. DAO is mainly expressed on the intestinal mucosa, and its level can be used as an indicator to assess the function of the intestinal barrier, where a high level indicates a damaged intestinal barrier [24]. This study revealed that there were higher quantities of *Alistipes* present within the gut microbiota of Han individuals compared with those in the Tibetan gut microbiota. *Alistipes* are positively correlated with IL-6 and intestinal permeability, and excessive amounts can damage the intestinal barrier, which further aggravates the risk of intestinal health of Han individuals [25]. In the Han population, the intestinal presence of 4-methylcatechol is notably elevated, potentially resulting in damage to the epithelial cells of the gastrointestinal tract [26].

In addition, the blood ALT level of the Han population was elevated, even though all research participants were healthy and had no history of liver disease. *Pseudomonas* was highly correlated with the blood ALT levels, likely due to *Pseudomonas* translocation from the intestinal tract [27]. Long-term high-altitude living increases the quantity of *Pseudomonas* within the gut of Han individuals, and the lower oxygen concentration at high altitude results in intestinal damage [28]. Three quarters of the blood supply to the liver is derived from intestinal tract back-bleeding. This enables *Pseudomonas* and toxins to release into the intestinal and hepatic circulation, which results in inflammatory reactions that can lead to liver lesions and injury [29].

In contrast, *Prevotella* was more common in the intestines of Tibetan people. *Prevotella* can regulate disordered intestinal microbiota, which results in strengthened barrier integrity, which, in turn, reduces the numbers of inflammatory molecules such as IL-6 and TNF-α [30]. *Prevotella* is capable of producing substantial quantities of ferulic acid [31]. This may account for the higher levels of ferulic acid found in the intestines of Tibetan individuals. Ferulic acid not only synergizes with SCFA to enhance the host’s intestinal integrity and immune function but also exerts protective effects on gut health by inhibiting the nuclear factor-κB pathway [26,27]. This mechanism contributes to the robust gut health observed in Tibetan populations, particularly in the challenging environment of the Qinghai-Tibet Plateau.

Hypoxia can induce overexpression of the TLR4/NF-κB pathway and release large numbers of inflammatory mediators to mediate the inflammatory response [32]. This phenomenon damages and increases the permeability of the capillary membrane of the lungs, brain, and other tissues, while also increasing the presence of inflammatory factors to upregulate cell adhesion molecules ICAM-1 and VCAM-1, which causes further leakage of capillaries and leads to high-altitude-related brain and pulmonary edema [33,34]. The frequency of cerebral edema and pulmonary edema manifestations is elevated among Han individuals dwelling on the Qinghai-Tibet Plateau. However, these incidences are very rare in the Tibetan population, which could stem from the elevated levels of pyrogallol in the gut of the Tibetan population, as pyrogallol can suppress ICAM-1 to reduce brain and pulmonary edema [35]. *Prevotella* and *Butyrivibrio* can produce various SCFAs [35], which can enter the brain through the blood–brain barrier [36], downregulate ICAM-1 and VCAM-1 levels, and reduce brain and pulmonary edema [37].

In this study, metabolomic analysis uncovered higher pyrogallol levels in the gut of Tibetans than those in Han individuals. Pyrogallol has anti-tumor proliferation and tumor cell apoptosis promotion effects [19]. Furthermore, the presence of a higher abundance of *Butyrivibrio* in the gut of Tibetan individuals exhibits a significant inhibitory effect on tumors [38].

Intense sunlight at high altitude may cause programmed cell death and cause skin tumors [39]; ferulic acid was present in higher abundance within the gut of Tibetan population, and this has a protective effect on the skin by improving the chemical stability of vitamins C and E, thereby enhancing the photoprotective effect [40]. In contrast, higher levels of 3-hydroxybutyric acid were found in the intestines of the Han population compared with those in the Tibetan population. The compound 3-hydroxybutyric acid is associated with adenomatous polyposis [41] and colorectal cancer [42]. The Han population exhibits a higher abundance of *Coprococcus* in the gut, which is highly correlated with the oncogene Agap2 [43].

The differences between the populations can be related to various factors. First, the genetic backgrounds (including the genetic composition and genetic diversity) of the populations differ, and genetics affect the intestinal microbial structure of the host [44]. Second, dietary habits are important factors affecting the intestinal microbial community. Dietary variations between the Tibetan and Han populations encompass distinctions in primary food sources and the distribution of intake proportions. The Tibetan people traditionally eat a high fiber, low-fat, plant-based diet [45], while the Han population diet is relatively diverse and includes both plant and animal food. The differences between the populations could be the result of interactions between these factors; genetic, dietary, and environmental factors are known to have complex interactions that may collectively influence the composition and metabolic characteristics of gut microbiota [46].

This study aids in understanding the adaptability and health status of different ethnic groups existing within the Tibetan Plateau environment. However, our study has certain limitations, such as the restricted number of participants and the horizontal study design. To further verify these findings, future studies could be conducted using a larger sample size and a vertical design.

## Figures and Tables

**Table 1:** Baseline information.

	Han (*n* = 39)	Tibetan (*n* = 40)
Age (mean ± SD)	40.34 ± 14.36	43.78 ± 18.60
Male (*n*)	17	18
Female (*n*)	22	22
BMI (mean ± SD)	23.0962 ± 2.9617	22.4409 ± 3.9102

**Table 2:** Alpha diversity analysis.

Index	*p*-Value	False Discovery Rate
Observed	0.019036	0.033289
Chao1	0.015531	0.033289
ACE	0.013325	0.033289
Coverage	0.022193	0.033289

**Table 3:** Differential metabolites in Tibetan and Han populations.

Peak	Han	Tibetan	*p*-Value
feces			
methanol phosphate	0.000365003	0.0007266	0.0247
serine minor	0.000396245	0.0003089	0.0157
isothreonic acid 4	0.000230432	0.0001756	0.0241
ferulic acid	0.000033955	0.0000752	0.0124
propane-1,3-diol	0.01722853	0.0141071	0.0049
linoleic acid methyl ester	0.000033922	0.0000753	0.0122
lyxose minor	0.001014129	0.0015144	0.0183
methylmalonic acid	0.000570163	0.0004681	0.0245
galactose-6-phosphate 2	0.000182541	0.0002707	0.0494
4-methylcatechol	0.000201333	0.0001691	0.0122
3-hydroxybutyric acid	0.001274244	0.0010258	0.0074
beta-hydroxymyristic acid	0.000150291	0.0002177	0.0461
pyrogallol	0.000006467	0.0000116	0.0005
pentonic acid	0.000366642	0.0002508	0.0057
melezitose	0.000491327	0.0003473	0.0493
serum			
methanol phosphate	0.0004135	0.0010335	0.0033
isothreonic acid 4	0.0003182	0.0002502	0.0447
ferulic acid	0.0000604	0.0001273	0.0324
linoleic acid methyl ester	0.0000603	0.0001273	0.0321
4-methylcatechol	0.0002778	0.0002414	0.0383
pentonic acid	0.0005072	0.0003565	0.0095
asparagine dehydrated	0.0008482	0.0006555	0.0159
fumaric acid	0.0004954	0.0003822	0.0119
2-methylglyceric acid NIST	0.0025448	0.0020593	0.0278

## Data Availability

The data used to support the results of this study can be found in the Supplementary Materials.

## Supplementary Materials

The following supporting information can be downloaded at: https://www.mdpi.com/article/10.3390/microorganisms11112655/s1, Table S1: Differences in β diversity; Table S2: Family composition of the Tibetan and Han individuals; Table S3: Species composition of the Tibetan and Han individuals; Table S4: The species that best explains the differences between groups; and Table S5: Differences in hematological parameters between the two groups.
